# Prevent Effects of *Lactobacillus Fermentum* HY01 on Dextran Sulfate Sodium-Induced Colitis in Mice

**DOI:** 10.3390/nu9060545

**Published:** 2017-05-25

**Authors:** Xiaoyong Chen, Xin Zhao, Hongwei Wang, Zelin Yang, Jian Li, Huayi Suo

**Affiliations:** 1 College of Food Science, Southwest University, Chongqing 400715, China; chenxiaoyong522@163.com (X.C.); wanghw_1978@swu.edu.cn (H.W.); 2 Chongqing Engineering Research Center of Regional Food, Chongqing 400715, China; 3 Chongqing Collaborative Innovation Center for Functional Food, Chongqing University of Education, Chongqing 400067, China; zhaoxin@cque.edu.cn; 4 Chongqing Proviencial center for Animal Disease Control and Precention, Chongqing 401120, China; yzl997@sina.com; 5 College of Life Science and Technology, Southwest University for Nationalities, Chengdu 610041, China

**Keywords:** traditional fermented yak yogurt, *Lactobacillus fermentum*, intestinal function, colitis, dextran sulfate sodium

## Abstract

The aim of this study is to assess the preventive effects of *Lactobacillus fermentum* HY01 (LF-HY01) to dextran sulfate sodium induced-colitis. We observed the ratio of colon weight to its length, colon pathological changes, and the concentrations of pro-inflammatory factors (IFN-γ, IL-12, TNFα, and IL-6) in serum. We also took account of the protein levels of IκBα, NF-κB p65, iNOS, and COX-2, and we measured the best effects of different doses of *Lactobacillus fermentum* HY01 (low dose group was 10^9^ CFU/kg·bw, high dose group was 10^10^ CFU/kg·bw) on dextran sulfate sodium-induced colitis mice. The results were remarkable, suggesting that *Lactobacillus fermentum* HY01 had significant preventive effects in dextran sulfate sodium induced-colitis; simultaneously, the high dose group showed the best results among other groups. It can effectively alleviate the shortened colon length, reduce the ratio of colon weight to its length, reduce edema, inflammatory cells infiltration, and colon mucosa injury, and play an important role in the down-regulation of concentrations of pro-inflammatory factors (IFN-γ, IL-12, TNFα, and IL-6). Above all, *Lactobacillus fermentum* HY01 shows promising prevention for IκBα degradation, inhibition of NF-κB p65 phosphorylation cascades, and decreases the protein levels of iNOS and COX-2 as well.

## 1. Introduction

Ulcerative colitis (UC) is a type of inflammatory bowel disease (IBD) [1] which is a non-specific chronic inflammation of the intestine including immune disorders, genetic predisposition, environmental factors, and certain lifestyle deprivations [2,3,4]. Common clinical symptoms are weight loss, abdominal pain, diarrhoea, and hematochezia. Although much progress has been made to categorize the complexity of IBD pathogenesis, its exact etiology is not clearly known, and there is no known cure currently available for this disease [5].

Dozens of different mouse models of colitis have been developed in order to study potential mechanisms of IBD pathogenesis and evaluate therapeutic effects. Among various different kinds of chemicals used to make colitis models, dextran sulfate sodium (DSS)-induced colitis is a widely used model because of its simplicity and many similarities with human UC. DSS is a highly water-soluble compound which is toxic for the colonic epithelial cells and causes defects in the epithelial barrier integrity, resulting in increased colonic epithelial permeability. Further, its anticoagulant property aggravates intestinal bleeding. Generally, DSS molecular weight is very critical for the severity of colitis; lower molecular weight (5 kDa) may result in milder colitis, while higher molecular weight (500 kDa) does not cause any injury to the colon. Therefore, a molecular weight of 36–50 kDa is used to induce colitis [6].

Probiotics can compete with pathogenic bacteria for mucosal adherence and simultaneously heal impaired mucosa; they can inhibit apoptosis, stimulate crypt cell proliferation, and regulate inflammatory cytokine expression as well as the intestinal immune system [7,8,9,10]. Therefore, the idea of using probiotics to cure IBD has attained specific importance. Numerous studies have demonstrated that the intestinal microbiota is significantly different between IBD patients/mice and healthy humans/mice; e.g., *Lactobacilli*, *Bifidobacterium*, *Streptococcus*, *Enterococcus*, *Fuso bacteria*, *Bacteroides* [11,12]. In addition, microbial dysbiosis in the gut can affect the synthesis and release of different inflammatory indicators, including pro-inflammatory cytokines such as tumor necrosis factor-α (TNF-α), IL-1β, Interferon-γ (IFN-γ), interleukin-1, 2, 6, 12 (IL-1, 2, 6, 12), anti-inflammatory cytokines (IL-4, IL-10), reactive oxygen and nitrogen metabolites, eicosanoids, and platelet activating factor [13].

Traditional fermented yak yogurt—a popular milk product in the Qinghai-Tibet plateau area, China—contains abundant lactic acid bacteria (LAB). We separated the dominant LAB strains from Xueo (traditional fermented yak milk brought from the western Sichuan Plateau of China); we took some genes from *Enterococcus durans*, *Lactobacillus fermentum*, and *Lactobacillus paracasei* using 16S rDNA sequence analysis and *atpA* gene analysis [14]. According to a survey of the composition of the microbiota in fermented yak milk at different Tibetan altitudes, *Lactobacillus fermentum* is the predominant lactic species in fermented milk [15]. Zhang showed that LAB dominate the kurut (naturally fermented yak milk) samples taken from the yaks of the plateau of the northwest and east of Qinghai, while yeast dominated in kurut samples from Huanhu yaks from the south of Qinghai [16]. In addition, there were six genera (*Lactobacillus*, *Lactococcus*, *Leuconostoc*, *Streptococcus*, *Enterococcus*, and *Weissella*) and twenty-one species of LAB existing in yak milk products in the Gansu Province of China. Due to the distinct climate conditions of the Qinghai-Tibet plateau, the living habits of herdsman, yak milk varieties, and fermentation conditions such as temperature and time, LAB from yak yoghurt is superior and contains many unique properties [17]. *Lactobacillus fermentum* Suo was isolated and extracted from yak yoghurt of Hongyuan (Sichuan, China), which has better qualities of acid-resistance, bile tolerance, hydrophobic property, and preventive effects, as well against activated carbon-induced constipation in mice [18]. *Lactobacillus helveticus* H9 was isolated from traditionally fermented yak milk in Tibet (China); it has the ability to produce the antihypertensive peptides Val-Pro-Pro and Ile-Pro-Pro during milk fermentation [19]. The results of the experiment in vitro showed that the two *Lactobacilli* strains (*Lactobacillus coryniformis* subsp. *torquens* T3L and *Lactobacillus paracasei* subsp. *paracasei* M5L) isolated from traditionally fermented milk exhibit immunomodulating capacity [20]. *Lactobacillus fermentum* HY01 (*LF*-HY01) was also isolated from traditionally fermented yak yoghurt. The aim of this study was to assess the preventive effects of *LF*-HY01 to colitis using DSS-induced colitis mice model.

## 2. Materials and Methods

### 2.1. Strain

*Lactobacillus fermentum* HY01 (LF-HY01) was isolated from traditional fermented yak yoghurt of Hongyuan grassland houseland (Ngawa Tibetan and Qiang Autonomous Prefecture, Aba, Sichuan, China), and it was deposited in the China Center for the Type Culture Collection (CCTCC, Wuhan, China), bearing CCTCC Accession Number M2015792.

### 2.2. Animals

Forty mice from Kunming (six weeks old) with half males and half females were purchased from the Experimental Animal Center of Chongqing Medical University (Chongqing, China). Animal permit number: SYXK (Yu) 2012–0001. The mice were exposed to the conditions (temperature 25 ± 2 °C, relative humidity 50% ± 5%, 12 h light/12 h dark cycle), and free access to a standard mice chow diet and water.

### 2.3. Isolated and Identified

LF-HY01 was isolated from traditional fermented yak yoghurt using the serial dilution and spread plate method and cultured at 37 °C for 48 h in MRS both (BD, Franklin Lakes, NJ, USA); the MRS solid medium contained 1.5% agar. The cell morphology was observed after gram staining, and the genus was analyzed using 16S rDNA sequence and phylogenetic tree. Firstly, the genome DNA from LF-HY01 was extracted by TIANamp bacteria DNA kit (Tiangen, Beijing, China), its 16S rDNA was amplified with S1000 Thermal Cycler (Bio-Rad, Hercules, CA, USA), and the amplification product was tested by using 1.5% agarose gel electrophoresis. Finally, the remaining amplification product was sent to sequence (BGI, Shenzhen, Guangdong, China). Finally, based on 16S rDNA sequence of LF-HY01 and reference strains, a phylogenetic tree was built by using MEGA 5.05. During the experiment, LF-HY01 was activated and cultured at 37 °C for 18 h in MRS both.

### 2.4. Survival Rate in Simulated Gastric Juice

Stimulated gastric juice (100 mL) was composed of pepsin (0.35 g) and NaCl (0.2 g). The pH value of the solution was adjusted to pH 3 with 1 mol/L HCl using a pH meter (pHS-3C, Shanghai Lei Ci Instrument Factory, Shanghai, China) and sterilized by filtering through a 0.45 µm filter (Millipore, Billerica, MA, USA). Ten-milliliter inoculums were centrifuged at 4000 r/min for 10 min, washed twice with 10 mL sterilizing saline (0.9%), then resuspended in the same buffer. One milliliter of the suspension was mixed with 9 mL stimulated gastric juice and incubated in a shaking incubator (37 °C, 150 r/min) for 3 h. After that, its colony forming units (CFU) was separately counted at 0 and 3 h. The survival rate (SR) was calculated using the following equation: SR (%) = (*N*_3_/*N*_0_) × 100, where SR is the survival rate of *Lactobacillus* and *N*_0_ is the number of viable cells (CFU/mL) at 0 h and *N*_3_ is the number of viable cells (CFU/mL) at 3 h [20].

### 2.5. Bile Salt Tolerance

One-hundred microliter inoculums were inoculated into 5 mL MRS-THIO (MRS broth plus 0.2% of sodium thioglycolate) with or without 0.3% (*w*/*v*) oxgall. Bacterial cell in the culture broth was measured by reading the optical density (OD) at 600 nm after 24 h incubation at 37 °C. The percentage of bile tolerance was calculated using the following equation: BT (%) = (*A*/*A*_0_) × 100, where BT is the bile tolerance of *Lactobacillus*, A is the OD of bacterial cell with 0.3% (*w*/*v*) oxgall, and N_0_ is the OD of bacterial cell without 0.3% (*w*/*v*) oxgall [20].

### 2.6. Induction of Colitis in Mice

To study the preventive effects of *Lactobacillus fermentum* HY01 against DSS-induced colitis, the animals were divided into four groups with five females and five males in each group. The experimental design was as follows: the normal group mice (N) was fed a normal diet and drank water from weeks 1 to 5. The model group (DSS) was fed a normal diet and drank water during the experiment, but drank water with 2% (*w*/*v*) DSS (molecular mass, 40 kDa; ICN Biomedicals, Aurora, OH, USA) in the third week and 4% DSS in the fifth week. The low- and high-dose groups were gavaged with 10^9^ CFU/kg·bw and 10^10^ CFU/kg·bw in weeks 1 to 5. Ten milliliter inocula were centrifuged at 4000 r/min for 10 min, washed twice with 10 mL 0.9% sterilizing saline and resuspended in 10 mL 0.9% sterilizing saline. We then determined colony counts by using plate colony-counting method and its absorbance at 600 nm by using the spectrophotometer. During this experiment, we used the results of colony counts and absorbance to adjust the certain concentration of LF-HY01 to gavage. The fresh inoculum was prepared once a day. In addition, low- and high-dose groups were fed a normal diet and drank water during the experiment, but drank water with 2% DSS in the third week and 4% DSS in the fifth week.

### 2.7. Collection of Samples

After 5 weeks, the mice were killed. We quickly measured the length and weight of the colon and took photos, then a portion of colon was used to make HE slice and the rest was stored at −80 °C after liquid nitrogen refrigeration to measure the levels of proteins. The serum was isolated (4 °C, 3000 r/min, 10 min) and stored at −80 °C.

### 2.8. Histological Observations

The colons were immediately removed after sacrificing and fixed in 10% (*v*/*v*) buffered formaldehyde, paraffin embedded, sectioned, followed by hematoxylin and eosin staining.

### 2.9. Measurement of Cytokine in the Serum

The concentrations of IFN-γ, IL-12, TNF-α, and IL-6 in the serum were measured by enzyme-linked immunosorbent assay kits (Beijing Bai Ao Lai Bo Co., Ltd., Beijing, China).

### 2.10. Western Blot Analysis

Proteins were extracted and analyzed by general Western blot protocol. Just for a brief period, proteins were extracted by RIPA buffer (R0010, Solarbio, Beijing, China) with 12,000 r/min centrifugation for 5 min and subjected to SDS-PAGE and transferred to 0.45 µm PVDF membranes (Millipore, Billerica, MA, USA). Membranes were blocked for 2 h at room temperature with 5% BSA, then incubated overnight at 4 °C with appropriate dilutions of primary antibodies (Abcam, Cambridge, UK), five washes with TBST, having 5 min each. This was then followed by incubation of membrane with conjugated secondary antibody (Abcam, Cambridge, UK) for 1 h at room temperature, and three washes with TBST at 5 min each. Finally,

### 2.11. Statistical Analysis

Data are presented as mean ± standard deviation, and the differences were evaluated using analysis of variance (ANOVA) with Duncan’s multiple range tests. *P* < 0.05 was considered statistically significant. The analysis was performed with SPSS 17.0 (IBM, Armonk, NY, USA).

## 3. Results

### 3.1. Strain

**Figure 1:**
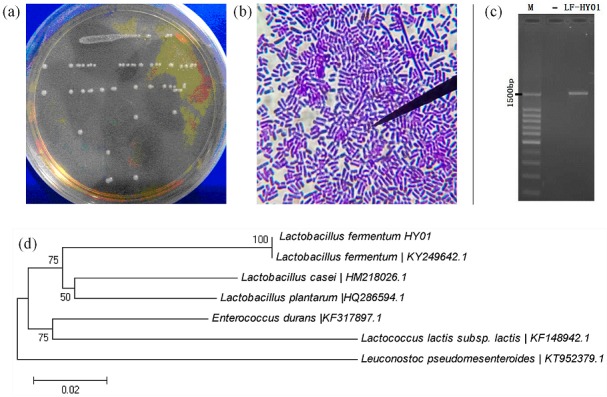
Isolation and identification of LF-HY01. (**a**) Colonial morphology of LF-HY01; (**b**) Cells form of LF-HY01; (**c**) Agarose gel electrophoresis of LF-HY01 16s rDNA PCR amplification product; (**d**) Phylogenetic analysis of LF-HY01.

LF-HY01 colonial morphology was consistent (Figure 1a), and cells were all rod shaped (Figure 1b). The electrophoresis result of 16S rDNA amplification product from LF-HY01 was satisfactory with clear band, correct place, and non-specific amplification (Figure 1c). After analyzing by 16S rDNA sequence and phylogenetic tree, the results of both the analyzing methods were consistent, and LF-HY01 was considered to be *Lactobacillus fermentum*. The specific results were that LF-HY01 had 100% homology with *Lactobacillus fermentum,* and LF-HY01 gathered together with *Lactobacillus fermentum* in the *Lactobacillus* branch (Figure 1d).

### 3.2. In Vitro Characteristics of Strain

The effect of stimulated gastric juice and different concentrations of bile salt can change the viability of LF-HY01 as presented in Table 1. LF-HY01 exhibited complete tolerance to simulated gastric juice, and its growth efficiency in bile salt decreased with the increase in bile salt concentration, but it still had its function in a very high level 0.3% bile salt as well.

### 3.3. Colon Weight and Length

**Figure 2:**
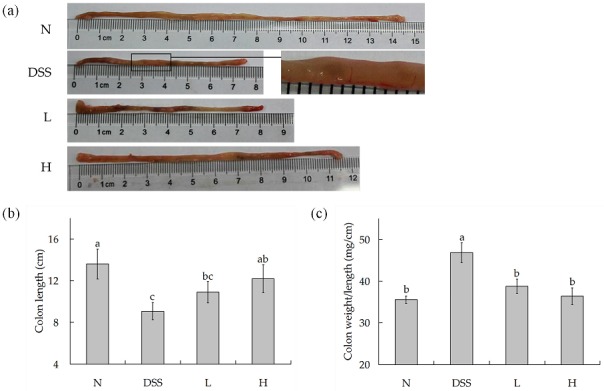
Colon length and weight of colitis mice. (**a**) Morphological observation of colon in mice; (**b**) Colon length in mice; (**c**) Colon weight/length in mice. *N* (normal group), dextran sulfate sodium (DSS, model group), *L* (low dose group, 10^9^ CFU/kg·bw), *H* (high dose group, 10^10^ CFU/kg·bw); ^a,b,c^ expressed as significant level, *p* < 0.05.

Shortening of the colon is one of the typical symptoms of colitis [21]. As shown in Figure 2a,b, the colon length was noticeably short in DSS-treated mice (9.08 ± 0.83 cm) than in normal mice (13.06 ± 1.43 cm). After being treated with different doses of LF-HY01, it was noticed that it had the ability to effectively alleviate the colon length, but the treatment had better effects with moderate dose. The colon length with low- and high-dose treatment was 10.90 ± 1.04 cm and 12.18 ± 1.33 cm, respectively. We know that edema is one of the typical symptoms of colitis [22]. The ratio of colon weight to colon length is generally used to indirectly understand edema extent (the larger the ratio, the more serious the edema). As shown in Figure 2c, the ratio of colon weight to colon length (mg/cm) was significantly increased in DSS-treated mice (46.84 ± 2.40 mg/cm) as compared to that of normal mice (35.55 ± 0.89 mg/cm). The ratio of different doses of LF-HY01-treated mice had a very significant difference with DSS-treated mice, but remarkably had a slight difference with normal mice, so it was proved that different doses of LF-HY01 treatment could remit the colon tissue edema.

### 3.4. Histological Analyses

**Figure 3:**
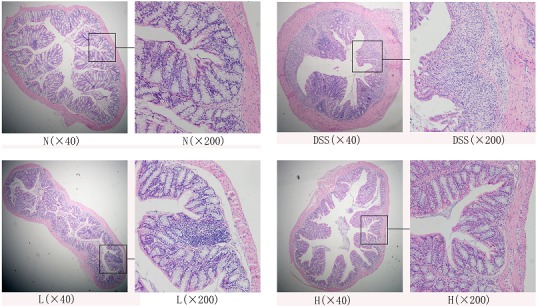
Histopathology observation of colon. N (normal group), DSS (model group), L (low dose group, 10^9^ CFU/kg·bw), H (high dose group, 10^10^ CFU/kg·bw).

The hematoxylin and eosin-stained sections were observed under microscope to evaluate the preventive effects in mice that were treated with LF-HY01 and had DSS-induced colitis. As is evident in Figure 3, the tissue sections of normal mice—as compared to that of the DSS-treated mice—showed colonic mucosal damage, decreased goblet cells, aberrant crypts, and considerable inflammatory cells infiltrations. However, the structure of tissue sections taken from LF-HY01-treated mice with different doses that had DSS-induced colitis was found almost normal; they had more intact colonic mucous epithelium, crypt glands, and less inflammatory reactions than those in the DSS-colitis mice. The results indicate that LF-HY01 can reduce colitis symptoms to a very great extent in DSS induced-colitis.

### 3.5. IL-6, IL-12, INF-γ, and TNF-α Concentrations in Serum

**Figure 4:**
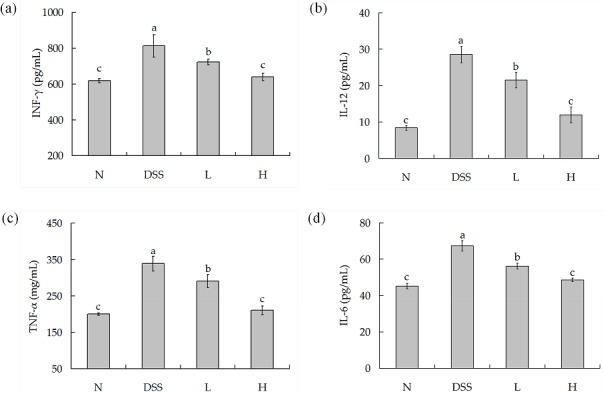
Pro-inflammatory factors concentrations in serum. (**a**) IL-6 cytokine level in serum; (**b**) IL-12 cytokine level in serum; (**c**) TNF-α cytokine level in serum; (**d**) IFN-γ cytokine level in serum. *N* (normal group), DSS (model group), *L* (low dose group, 10^9^ CFU/kg·bw), *H* (high dose group, 10^10^ CFU/kg·bw); ^a,b,c^ expressed as significant level, *p* < 0.05. TNF-α: Tumor necrosis factor-α; IFN-γ: interferon-γ; IL-6, 12: interleukin-6, 12.

Cytokines are low molecular weight proteins which can induce or regulate a variety of immune or inflammatory responses, and play a critical role as a host. IL-6, IL-12, INF-γ, and TNF-α are all pro-inflammatory [23]. As shown in Figure 4a–d, the IL-6, IL-12, INF-γ, and TNF-α concentrations of normal mice were lowest. The concentrations of these cytokines were highest in the DSS-colitis mice. All these cytokine concentrations in serum were significantly decreased after the treatment of different doses of LF-HY01—especially the moderate dose, which did not differ much from the normal mice.

### 3.6. Western Blot

**Figure 5:**
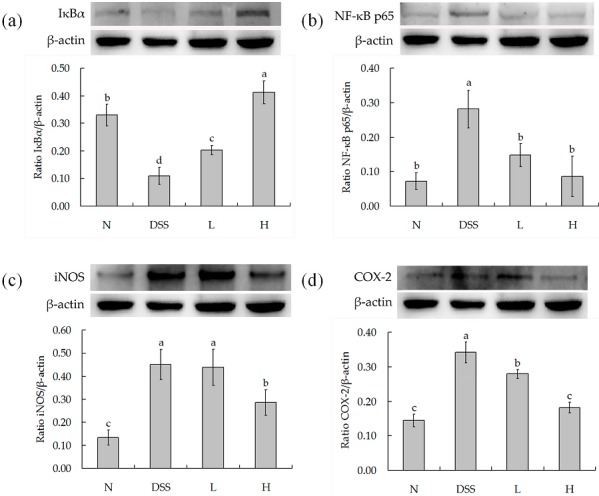
Protein levels in colon. (**a**) Semiquantitative analysis of protein ratio IκBα/β-actin; (**b**) Semiquantitative analysis of protein ratio NF-κB p65/β-actin; (**c**) Semiquantitative analysis of protein ratio iNOS/β-actin; (**d**) Semiquantitative analysis of protein ratio COX-2/β-actin. *N* (normal group), DSS (model group), *L* (low dose group, 10^9^ CFU/kg·bw), *H* (high dose group, 10^10^ CFU/kg·bw); ^a,b,c^ expressed as significant level, *p* < 0.05.

IκBα, NF-κB p65, iNOS, and COX-2 as determined by Western blot are shown in Figure 5a–d. DSS treatment concentration could significantly decrease the level of IκBα and increase the level of NF-κB p65, iNOS, and COX-2. However, LF-HY01 could increase the level of IΚBα and decrease the levels of NF-κB p65, iNOS, and COX-2 in DSS-colitis mice.

## 4. Discussion

Probiotics are recognized as live microorganisms which confer health benefit to the host when administered in adequate amount, so as to maintain the balance of the intestinal microbiota and enhance immunity [24]. Most probiotic strains are *Lactobacilli* which were isolated from infant, healthy adult, or fermented foods, generally evaluated by different selection criteria, which include: withstand transit through the gastrointestinal tract, the capacity to inhibit intestinal pathogens, the ability to colonize the intestinal tract, health benefits, etc. However, only probiotic strains surviving through the passage of the stomach and the small intestine have full scope to give the health benefits to the host [25,26]. Therefore, resisting gastric juice and bile salts in the gastrointestinal tract is the fundamental requirement [18]. In this study, based on an external virtual model of the gastrointestinal tract, we defined that LF-HY01 was not affected at pH 3.0 simulated gastric juice, and also had the ability to keep good growth efficiency in different concentrations of bile salts. This indicates that LF-HY01 was suitable for the study purpose based on functional effects associated with health benefits.

Generally speaking, colon length, colon edema, and mucosal damage are useful indices to evaluate the degree of inflammation; they are typical symptoms of colitis. When the colon length is shorter, the symptoms (colon edema, mucosal damage) are also more serious, and the most severe is inflammation [21,27]. For example, shortening of the colon, edema, and bleeding are macroscopic appearances in the TNBS-induced colitis mouse model [28]. In the same way, the macroscopic appearances were same in the DSS-induced colitis mouse model [29]. In this study, we observed shortening of the colon, edema, and bleeding after the injuries induced by DSS, and treatment with LF-HY01 could not only relieve shortened colon and reduce the ratio of colonic weight to length, but also prevented mucosal damage, helped to increase goblet cells, and remitted inflammatory cells infiltration. Therefore, we speculated that LF-HY01 may improve the healthy functioning of the intestinal mucosa.

Cytokines play an important role in the modulation of the immune system, which contains anti-inflammatory cytokines factors and pro-inflammatory cytokines factors in the inflammatory response, and which are related to Th1 or Th2 cells. Orchestrating cell-mediated immunity is the key ability of Th1 cells, and they can secrete INF-γ, IL-2, and IL-12. In contrast, mediating humoral responses is the key ability of Th2 cells, and they can secrete IL-4, IL-5, IL-6, IL-10, and IL-13. These subsets can regulate each other reciprocally through key cytokines. INF-γ can suppress the development of Th2, whereas IL-4, IL-10, and IL-13 secreted by Th2 cells inhibit Th1 responses, and the effect of regulating each other maintains normal state. During the period of inflammation, this adjustable effect was damaged, hence promoting inflammation. INF-γ, IL-12, TNF-α, and IL-6 are all pro-inflammatory cytokines which can cause immune disorders and amplify the inflammation. INF-γ is a dimeric glycoprotein, and it can badly affect the structure and function of intestinal epithelial cells and increase the permeability of intestinal mucosa [30]. IL-12 can not only promote the differentiation of Th0 cells into Th1 cells and the production of IL-2 and IFN-γ, it can also inhibit the differentiation of Th0 cells into Th2 cells and the synthesis of IgE [28]. TNF-α is the earliest and most important inflammatory mediator; it can gather inflammatory cells and lead to inflammatory cells infiltration and tissue edema [31]. IL-6 can promote the synthesis of acute protein and gather T cells in inflammatory sites [32]. In this study, we demonstrated that IFN-γ, IL-12, TNF-α, and IL-6 increase in DSS-induced colitis mice. However, the concentrations of IFN-γ, IL-12, TNF-α, and IL-6 decrease after LF-HY01 treatment.

In addition, probiotics could inhibit activated NF-κB [33]. NF-κB is a nuclear transcription factor which can regulate many genes, apoptosis, viral replication, tumorigenesis, and inflammation. NF-κB was normally inactive in the form of p50-p65-IκBα, because it was inhibited by IκBα. However, under some conditions (growth factors, cytokines, lymphokines, UV, pharmacological agents, and stress), IκBα is ubiquitinated and phosphorylated, causing the activation of NF-κB. This result would accentuate inflammation with the creation of adhesion molecules, transcription factors, inflammatory cytokines, and cell surface receptors in the end. Furthermore, NF-κB has the transcription regulation genes iNOS and COX-2, so it can regulate the expression of iNOS and COX-2 inflammatory proteins. When NF-κB is activated, the protein levels of iNOS and COX-2 increase. iNOS is a messenger molecule with diverse functions throughout the body; it can produce nitric oxide (NO), which has the ability to relax blood vessels and raise the vessel permeability. Thus, it has advantages of the inflammation medium to reach inflammatory sites and leads to inflammation aggravation. COX-2 is an inducible enzyme which is hardly expressed in normal body, but its level is up-regulated in response to body damage during colitis. COX-2 is known to synthesize prostaglandins (PGs) from arachidonic acid, which is associated with the mediation of colitis. Furthermore, it has been reported that the expression levels of COX-2 and PGE2 were elevated in the inflamed mucosal tissues of patients/mice with colitis. Above all, these results eventually promoted the secretion of intestinal epithelial cells, caused edema, expanded blood vessels, and increased permeability of the intestinal mucosa [23]. In this study, we used DSS-induced colitis, and we observed an increase in NF-κB p65 protein levels and a decrease in IκBα protein levels. Moreover, we also found the protein levels of iNOS and COX-2 increased. After LF-HY01treatment, we observed a decrease in NF-κB p65 protein levels and an increase in IκBα protein levels, and we also found reduced protein levels of iNOS and COX-2. It has been shown that LF-HY01 directly inhibited the expression of NF-κB-dependent cytokines by suppressing the activators of pro-inflammatory cytokines (iNOS and COX-2) [23], and thus reduced inflammation. Previous studies showed that probiotics can significantly improve intestinal immune function, so probiotics can directly or indirectly regulate intestinal immune response [11]. *Lactobacillus fermentum* 5716 can reduce TNF-α levels and iNOS expression in the TNBS model [34]. *Lactobacillus brevis* HY7401 and *Lactobacillus plantarum* HY115 could inhibit colon shortening, suppressed the mRNA expressions of IL-1β, IFN-γ, and TNF-α, and also the protein levels of IL-1β and IL-6 in the colon of DSS-induced colitic mice. Therefore, these results suggested LF-HY01 could improve colitis in this study. In this study, LF-HY01 could prevent IκBα degradation, inhibit NF-κB p65 phosphorylation cascades, and reduce the protein levels of iNOS and COX-2. Therefore, LF-HY01 had the advantages of preventing the effects of colitis. However, how to regulate immune response of LF-HY01 is still unclear, so it is very important to further evaluate its mechanism of preventing colitis, because this can provide the basic theories for the functional application of LF-HY01.

## 5. Conclusions

*Lactobacillus fermentum* has been known as a probiotic. *Lactobacillus fermentum* HY01 (LF-HY01) which was isolated from traditional fermented yak yoghurt had good acid resistance, bile tolerance, and preventive effects for colitis. The preventive effects included improving colon cells as well as length damage, lowering the concentrations of pro-inflammatory factors (INF-γ, IL-12, TNF-α, and IL-6), increasing the protein levels of IκBα, and decreasing the protein levels of NF-κB p65, iNOS, and COX-2.

## Figures and Tables

**Table 1:** Effect of simulated gastric juice and bile salt on viability of LF-HY01.

Strain	Survival in PH 3.0 Simulated Gastric Juice (%)	Growth Efficiency in Bile Salt (%)			
		0.05	0.1	0.2	0.3
LF-HY01	103.73 ± 8.60	98.30 + 0.09	61.50 ± 0.45	29.40 ± 0.80	21.62 ± 0.86
